# High-Level Production, Solubilization and Purification of Synthetic Human GPCR Chemokine Receptors CCR5, CCR3, CXCR4 and CX3CR1

**DOI:** 10.1371/journal.pone.0004509

**Published:** 2009-02-18

**Authors:** Hui Ren, Daoyong Yu, Baosheng Ge, Brian Cook, Zhinan Xu, Shuguang Zhang

**Affiliations:** 1 Center for Biomedical Engineering, Massachusetts Institute of Technology, Cambridge, Massachusetts, United States of America; 2 Center for Bioengineering and Biotechnology, China University of Petroleum, Qingdao, Shandong, People's Republic of China; Instituto de Tecnologia Química e Biológica, Portugal

## Abstract

Chemokine receptors belong to a class of integral membrane G-protein coupled receptors (GPCRs) and are responsible for transmitting signals from the extracellular environment. However, the structural changes in the receptor, connecting ligand binding to G-protein activation, remain elusive for most GPCRs due to the difficulty to produce them for structural and functional studies. We here report high-level production in *E.coli* of 4 human GPCRs, namely chemokine receptors (hCRs) CCR5, CCR3, CXCR4 and CX3CR1 that are directly involved in HIV-1 infection, asthma and cancer metastasis. The synthetic genes of CCR5, CCR3, CXCR4 and CX3CR1 were synthesized using a two-step assembly/amplification PCR method and inserted into two different kinds of expression systems. After systematic screening of growth conditions and host strains, TB medium was selected for expression of pEXP-hCRs. The low copy number pBAD-DEST49 plasmid, with a moderately strong promoter tightly regulated by L-arabinose, proved helpful for reducing toxicity of expressed membrane proteins. The synthetic Trx-hCR fusion genes in the pBAD-DEST49 vector were expressed at high levels in the Top10 strain. After a systematic screen of 96 detergents, the zwitterionic detergents of the Fos-choline series (FC9-FC16) emerged as the most effective for isolation of the hCRs. The FC14 was selected both for solubilization from bacterial lysates and for stabilization of the Trx-hCRs during purification. Thus, the FC-14 solubilized Trx-hCRs could be purified using size exclusion chromatography as monomers and dimers with the correct apparent MW and their alpha-helical content determined by circular dichroism. The identity of two of the expressed hCRs (CCR3 and CCR5) was confirmed using immunoblots using specific monoclonal antibodies. After optimization of expression systems and detergent-mediated purification procedures, we achieved large-scale, high-level production of 4 human GPCR chemokine receptor in a two-step purification, yielding milligram quantities of CCR5, CCR3, CXCR4 and CX3CR1 for biochemical, biophysical and structural analysis.

## Introduction

G-protein-coupled receptors (GPCRs) primarily function as cell-surface receptors responsible for the transduction of extra-cellular stimuli into intra-cellular signals by binding extra-cellular ligands including photons, ions, lipids, peptides, nucleosides, nucleotides, neurotransmitters and peptide hormones. Structurally, they share a common hydrophobic core composed of seven-transmembrane α-helices (7TM) [1], [2]. Approximately 4% of human genes code for GPCRs and by the current count there are ∼800 functional genes. They comprise the largest superfamily of human integral membrane proteins [3], [4]. GPCRs play vital roles in a wide range of biological processes and are involved in a remarkable array of signaling events ranging from memory, sight, and smell to sexual development and the regulation of blood pressure [5], [6]. Therefore, GPCRs are attractive therapeutic targets for drug design. Currently, about 50% of pharmaceutical drugs target GPCRs [3]. Despite their critical importance, our current understanding of structure and function of GPCRs is inadequate because of their low natural abundance. Thus, for structural studies, which require milligram quantities of purified membrane protein [7], production in heterologous systems is required, but has been extremely difficult to accomplish. Up to now the molecular structures of only 5 unique GPCRs have been determined including bovine rhodopsin with and without the retinal ligand as well as with a C-terminal 11-residue peptide fragment of a Gα-protein (Gα-CT) [8], [9], [10]; a highly engineered human β_2_-adrenergic receptor with a replaced intracellular loop 3 (IC3) [11], [12], and a turkey β_1_-adrenergic receptor with the IC3 domain partly removed and most C-terminus deleted [13]. Currently not a single chemokine receptor structure is known.

Determination of the molecular structures of GPCRs including chemokine receptor still remains an enormous challenge, largely due to the notorious difficulty to obtain large quantities of purified proteins. The same is true for other membrane proteins. This is evident also from the fact that there are only 178 unique membrane protein structures among 410 membrane protein structures from over 54,000 structures available in the current Protein Data Bank http://www.rcsb.org/pdb/home/home.do (November 2008).

For over 50% of these determined membrane protein structures, the proteins were purified from naturally abundant sources. In contrast, less than 10% of soluble proteins were from natural sources, and over 90% were produced as recombinant proteins [14]. Therefore, future efforts need to focus on procedures for high-level heterologous expression of membrane proteins, effective solubilization in the presence of surfactants and purification for crystallization screening [15], [16].

Heterologous expression of functional GPCRs has been accomplished in *Escherichia coli*, yeast, insect and mammalian cells, but with varying degree of success because of differences in host cell environment [17], [18]. Insect and mammalian cell expression systems have been most frequently employed for pharmacological development. For biophysical and structural studies that require large amounts of GPCRs, yeast and *E. coli* systems are attractive for their ease of large-scale production but have been used with varying success [7].

There is no universal system suitable for GPCR production. The approach to achieving high-level production must often rely on empirical solutions for each particular GPCR. *E.coli* is a widely used system for heterologous protein production and is often perceived as an easy way to produce large amounts of eukaryotic proteins because of its simplicity of use and the availability of various expression plasmids and *E.coli* strains which have been reported to support high-level protein production. Furthermore, the short time required for plasmid construction and expression allows rapid optimization of purification schemes and inexpensive material for purification [7], [17], [19].

However, reports of GPCR expression in *E.coli* have shown extremely low yields [20]. Many factors may affect the efficiency including 1) codon usage efficiency, 2) translational initiation, 3) mRNA stability, 4) stability of the expressed protein, and 5) toxicity of the expressed protein in the host cells [7]. Several methods have been developed to overcome these problems and increase the protein yield. Notably, fusing target proteins to a highly expressed bacterial protein has proven particularly effective for improving the expression level of membrane proteins in *E. coli* [21]. Several GPCRs have been functionally expressed in *E.coli* and purified in milligram quantities as fusion proteins [22], [23], [24].

Solubilization and stabilization of membrane proteins using detergents are the first critical steps in purification membrane proteins, and constitute a bottleneck for the structural biology of membrane proteins [25], [26]. Membrane proteins require a membrane-like environment to maintain their correctly folded structures and functions during and after purification. Detergent micelles provide such environments surrounding the hydrophobic domains of membrane protein and keeping them soluble in an aqueous environment, and thus are widely used for solubilization and purification of membrane proteins [27]. Generally, the suitable detergent or detergent mixture should solubilize the target protein most effective, keep it stable, and prevent its self-aggregation. However, due to individual differences between membrane proteins, the choice of detergent or detergent mixture for a particular protein cannot be predicted. Therefore, a systematic approach is required to select the optimal detergents to achieve solubilization and stabilization of each target protein.

Chemokines are a family of small chemotactic cytokines (∼8–14 kDa), which function as chemo-attractants for various types of leukocytes and play a vital role in host defense mechanisms and lymphocyte development [6], [28], [29], [30], [31]. Chemokines are divided into four subfamilies based on the arrangement of two N-terminally conserved cysteine residues: α- or CXC chemokines (recently named CXC ligands, CXCL), β- or CC chemokines (CCL), γ- or C chemokines (lymphotactin, XCL) and CX3C chemokines (fractalkine, CX3CL) [6], [29], [30], [31]. The biological functions of chemokines are mediated by binding to cell surface chemokine receptors, which belong to the superfamily of G-protein-coupled receptors (GPCRs) [29]. Chemokines and their receptors are implicated in a wide range of human diseases, including acute respiratory distress syndrome, allergic asthma, psoriasis, arthritis, multiple sclerosis, cancer metastases, atherosclerosis, and AIDS infection. [6], [28], [32], [33]. We selected 4 human chemokine receptors in this study.

Human chemokine receptors CXCR4 (hCXCR4, termed Fusin) and CCR5 (hCCR5) have been identified as principal co-receptors, besides CD4, for entry of human immunodeficiency virus 1 isolate (HIV-1) into target cells. Specifically, CXCR4 is used for T cell line-tropic strains (X4), CCR5 for macrophage-tropic strains (R5), and another HIV-1 strains (R5X4), also named dual-tropic primary isolate, utilizes both CXCR4 and CCR5 as entry co-receptor [34], [35]. Additionally, CXCR4 and its specific ligand SDF-1 were shown to be associated with several cancers, such as breast cancer, head and neck cancer, small-cell lung cancer and non-small-cell lung cancer [36], [37]. Human chemokine receptor CCR3 (hCCR3) is highly expressed on eosinophils and binds multiple chemokine ligands such as eotaxin (CCL11), eotaxin-2 (CCL24), eotaxin-3 (CCL26), and MCP-4 (CCL13) with high affinity [38]. CCR3 also function as co-receptor for some isolates of HIV-1 and HIV-2 [39]. Furthermore several clinical studies suggest that eotaxin/CCR3 plays a pivotal role in allergic diseases, including allergic asthma, rhinitis, and atopic dermatitis [31], [40].

Human chemokine receptor CX3CR1 (hCX3CR1), the specific receptor for the fractalkine (FKN), is expressed on inflammatory leukocytes such as natural killer cells, monocytes and T lymphocytes, and mediates both cell-adhesive and migratory behavior of leukocytes, in addition, it is expressed at particularly high levels in neurons and microglia in the brain [41], [42]. CX3CR1 was also identified as a co-receptor together with CD4 for entry of HIV-1 [43], and a role for CX3CR1-FKN-mediated inflammation has been suggested in various inflammatory diseases including vascular injury, atopic dermatitis and allergic airway diseases [44].

Altogether, these 4 chemokine receptors represent attractive targets for intervention against such major diseases as AIDS, cancers and allergic diseases. Recently, certain chemokine receptor antagonists derived from chemokine peptides and small molecules were developed to block chemokine receptors [45]. However, the mechanisms of receptor interaction with their ligand and drugs remain poorly understood for lack of detailed molecular structures of chemokine receptors.

Here we report high-level productions of human GPCR chemokine receptors CCR5, CCR3, CXCR4 and CX3CR1. We synthesized the genes using a two-step assembly/amplification PCR method, then inserted them into pEXP3-DEST and pBAD-DEST49 vectors for high-level expression screening in *E.coli*. We first systematically screened and selected optimal: 1) *E.coli* host strains, 2) growth media, 3) induction and 4) temperature for protein production. After optimizing the expression of these chemokine receptors, we proceeded to systematically screen 96 detergents for use in purification procedures. The Fos-choline series, particularly FC14, was found to be most effective. This finding corroborates the previous work by Cook et al., who screened a wide range of detergents and found FC14 to be the optimal detergent for solubilizing and stabilizing another human GPCR, olfactory receptor hOR17-4 [46]. We also carried out secondary structural analysis of the purified protein and confirmed their molecular identities using monoclonal antibodies and some cases, mass spectroscopy analysis. To our knowledge, this is the first high-level production of human GPCR chemokine receptors in *E.coli* in milligram quantities sufficient for initiating structural studies.

## Results and Discussion

### PCR-based gene synthesis of human chemokine receptors

**Figure 1:**
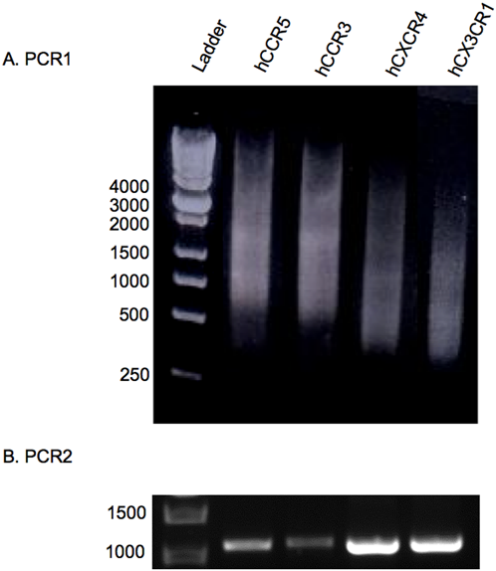
Analysis of PCR-based gene synthesis of human chemokine receptors. Full-length genes of hCCR5 (1,056 bp), hCCR3 (1,065 bp), hCXCR4 (1,056 bp) and hCX3CR1 (1,065 bp) were *de novo* synthesized using a two-step assembly/amplification PCR method and examined on 1% TAE agarose gel electrophoresis by staining with 2 µg/ml of ethidium bromide (EB). Samples (5 µl) were mixed with 1 µl of 6× DNA loading buffer per lane. 1 Kb DNA ladder is also shown. A) Detection of assembly PCR (PCR1) products of each hCR. B) Detection of amplification PCR (PCR2) products of the full-length genes from PCR1.

Genes of hCCR5, hCCR3, hCXCR4 and hCX3CR1 were *de novo* synthesized from a set of overlapping DNA oligonucleotides using a two-step assembly/amplification PCR method [47]. Using the online program DNAworks http://helixweb.nih.gov/dnaworks [48], the gene sequences of hCCR5, hCCR3, hCXCR4 and hCX3CR1 were optimized using human codon preference (Figure S1). The full-length genes were parsed into 42 oligos with the maximum length of 45 nucleotides. Assembly PCR (PCR1) and amplification PCR (PCR2) were successfully carried out (Figure 1). The PCR1 products (Fig 1A) including the full-length genes were later specifically amplified in PCR2 (Fig 1B). The number of PCR cycles in PCR1 is very important for successfully producing the full-length gene fragments. This number in some degree depends on the number of oligonucleotides in the starting set, as was described by Baedeker [49]. However, this still is an empirical approach to obtain the best results for each gene. Subsequently, we cloned the 4 synthetic hCR genes into pCT-Blunt II-TOPO vector by TOPO Cloning. In the same manner, our group also successfully synthesized several genes including human tetraspanin membrane protein CD81 and human olfactory receptor 17-4, mouse olfactory receptors mOR23, mS51 and I7 [46], [50], [51].

### The choice of expression vector systems

Synthetic genes of hCRs were expressed in *E.coli* system using Gateway® Technology since it is the most commonly used system for heterologous protein productions. It is also convenient for plasmid construction and optimization of expression levels [7]. For rapid testing of protein expression and efficient purification, a His_6_-tag was fused to the C-terminus of the hCRs in the *att*B-PCR step. Finally, each synthetic hCR gene was independently subcloned into pEXP3-DEST vector and pBAD-DEST49 vector, for screening different expression systems.

**Figure 2:**
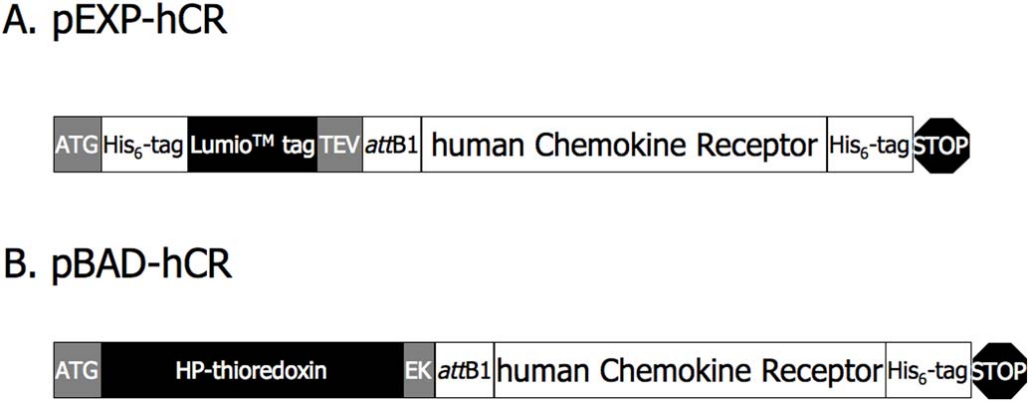
Schematic illustrations of 4 hCRs expressed as fusion proteins. In each synthetic hCR gene with a His_6_-tag was inserted at the C-terminus for expression, detection and protein purification, and each gene was cloned into pEXP3-DEST and pBAD-DEST49 vectors, respectively. A) Schematic representation of hCR cloned into pEXP3-DEST vector (simply pEXP-hCR). The translation start codon (ATG) followed by His_6_-tag, Lumio™ –tag and TEV (TEV protease recognition site) are present in the vector pEXP3-DEST and fused at the N-terminus of hCR following the insertion of the C-terminally His_6_-tagged hCR at the *att*B1 site by the Gateway site-specific recombination reaction. B) Schematic representation of hCR cloned into pBAD-DEST49 vector (pBAD-hCR). Analogous to the pEXP-hCR vector, the ATG, HP-thioredoxin (Trx) and EK (Enterokinase recognition site) are present in the pBAD-DEST49 vector and fused at the *att*B1 site to the N-terminus of hCR, following Gateway protocols.

The high-copy-number pEXP3-DEST plasmid vector, which has a strong T7 promoter, is designed for *in vitro* protein production although it is also useful for expression in *E.coli* strains expressing the T7 RNA polymerase. The elements of hCR constructs in the pEXP3-DEST (pEXP-hCR) include a N-terminal His_6_-tag and a Lumio tag followed by a TEV recognition site, and an *att*B1, flanked fragment inserted by the Gateway Cloning technique (Figure 2A).

The arabinose P_BAD_ promoters, which provide tight, dose-dependent regulation of heterologous gene expression and inducible with L-arabinose, are particularly suitable for expression of membrane proteins [52]. The low-copy-number pBAD-DEST49 plasmid vector was used as a Gateway Cloning destination vector for the synthetic hCR genes. They feature the fusion partner HP-thioredoxin (Trx-), followed by an Enterokinase recognition site (EK) and *att*B-flanked fragment containing the gene of interest (Figure 2B). The fusion partner HP-thioredoxin (His-Patch thioredoxin) is modified from *E.coli* thioredoxin by E32H and Q64H mutations to form a “patch” together with His_8_, which binds metal ions, to purify proteins on metal-chelating resins. Furthermore, thioredoxin functions as a translation leader for high-level expression and in some cases, to improve the solubility and stability of the fused membrane proteins [53].

### Selections of *E.coli* host strains

Unlike soluble proteins, membrane proteins, in general, have to be inserted into host membranes to assume their correct conformation. This often introduces toxicity to host cells during overexpression. Several *E.coli* strains have been developed to reduce such toxic effects associated with high-level expression of membrane proteins. However, the choice of the most suitable host strains for expression of a particular membrane protein is still empirical. Therefore, to attain high-level production, milligrams per liter of cell culture of functional membrane proteins, a systematic optimization of the expression system need to be carried out in a combinatorial manner including: 1) transcription promoters, 2) expression vectors, 3) host cell strains, 4) growth media conditions, 5) inducer concentration, 6) timing of induction, 7) duration of post induction, and 8) growth temperature of post induction [7].

Host strain screening for expression of pEXP-hCRs, was carried out with 5 *E.coli* strains: 1) BL21(DE3)-Star-pLysS (BL21), 2) C41(DE3) (C41), 3) C43(DE3) (C43), 4) C41(DE3)-pLysS (C41pLysS) and 5) C43(DE3)-pLysS (C43pLysS). The strain C41 and C43 were derived from BL21 to achieve high-level production of heterologous membrane proteins, reportedly for solving the problem of plasmid instability during the expression of toxic recombinant membrane proteins [54], [55].

**Figure 3:**
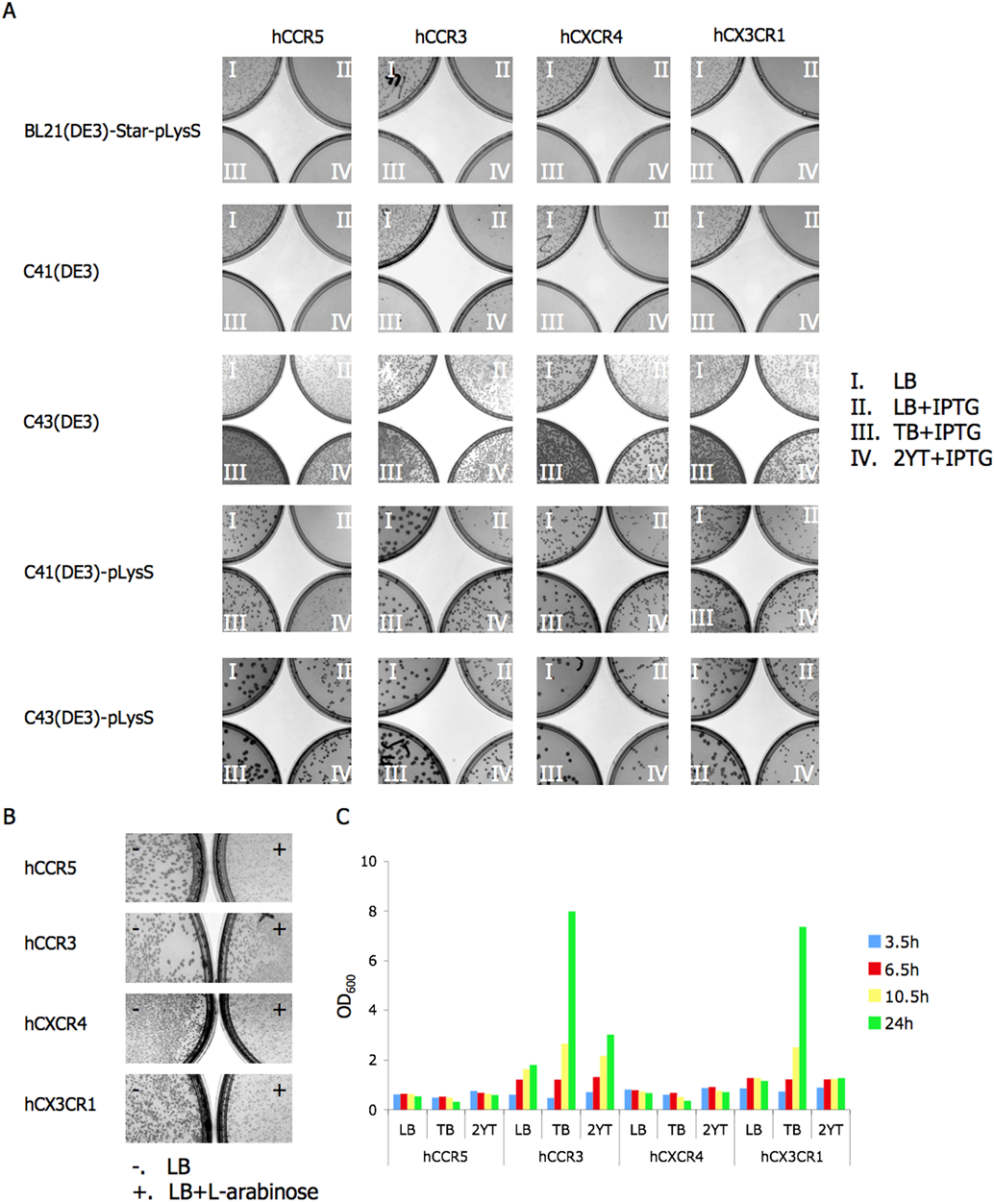
Selection of *E.coli* host strains and media for inducible expression of hCRs. A) Screening of *E.coli* strains and media for pEXP-hCRs. Each of pEXP-hCRs was transformed into all 5 *E.coli* strains: BL21, C41, C43, C41-pLysS and C43-pLysS, and spread on LB-agar plates (I), LB-agar-IPTG plates (II), TB-agar-IPTG plates (III) and 2YT-agar-IPTG plates (IV) for colony analysis. B) L-arabinose induction pBAD-hCRs for *E.coli* strain Top10. LB-agar plates without (−) and with (+) L-arabinose (0.05%) were used. C) Growth conditions of pBAD-hCRs in Top10 in the presence of inducer. Cell culture density at 600 nm (OD_600_) after indicated induction time, 3.5 hours, 6.5 hours, 10.5 hours and 24 hours, was measured.

We compared LB-agar plates I to II, BL21 and C41 cells that cannot form colonies in the presence of inducer IPTG (II), typical for expression of membrane proteins (Figure 3A). However, it does not necessarily suggest that the foreign gene is toxic to host strains since the conditions for growth and induction may also influence the expression level. C43, C41pLysS and C43pLysS were able to form smaller colonies in the presence of IPTG (II) than those of in the absence of IPTG (I), which, although a promising sign, does not always guarantee successful expression of target genes [56]. Good growth was observed for pBAD-hCRs in Top10, which is the strain recommended in the manufacturers protocol since its deficient in *ara*BADC. In the presence of L-arabinose (plates denoted with a +), Top10 cells formed colonies with smaller size than those on plates lacking L-arabinose (−) (Figure 3B).

The expression levels of pEXP-hCRs in each of the 5 strains and of pBAD-hCRs in Top10 were studied from 5 selected colonies, each picked from the plates without inducer. The results suggested that pEXP3-hCCR3, hCXCR4 and hCX3CR1 were best expressed in C41 strain, to some extent, and expressed at a lower level, in C43 and BL21, but not expressed well in C41pLysS and C43pLysS. For the pEXP-hCCR5 construct, detectable expression was not achieved in any of these strains. Expression was in all cases induced using 0.25 mM IPTG in TB medium at 16–18°C for 12–20 hours with shaking. The colonies of pEXP-hCRs in C43, C41pLysS and C43pLysS were larger than those in C41 and BL21 (Fig 3A). However, the gene expression levels in the former three strains appears to be lower than in the latter two strains. The, choice of the C41 over the BL21 strain for expression of pEXP-hCRs, was motivated by its better resistance to the toxic effects of heterologous membrane proteins in the host cells, as was described [54]. The expression of pEXP-hCRs in C41 was later studied by Western blot using mouse His_6_-tag monoclonal antibody.

### Selection of growth media

The choice of growth media has a significant influence on cell growth and protein production. Thus, selection of suitable growth media and conditions for pEXP-hCR expression in each of the 5 *E.coli* strains was carried out by comparison of colonies between plates II, III and IV (Figure 3A). TB (Terrific Broth) medium plates (III) showed better growth conditions than other two types of media. For testing growth conditions for pBAD-hCRs in Top10 (Figure 3C), cell growth was further studied in liquid media. The culture density of pBAD-hCCR3 and pBAD-hCX3CR1 in Top10 significantly increased in TB medium as function of time, but no obvious change was observed for hCCR5 and hCXCR4. The cell density of pBAD-hCCR3 and hCX3CR1 in Top10 was able to reach OD_600_ 7–8 units while those of hCCR5 and hCXCR4 only reached OD_600_ 0.6–0.8, a 10-fold difference. The expression of hCCR5 and hCXCR4 thus appeared to induce a stronger toxic response in the host cells than hCCR3 and hCX3CR1.

It is not surprising that different proteins have individual characteristics and behave differently in identical cells, which is a common observation in heterologous membrane protein expression [7]. It is possible that media rich in nutrients in some cases may contribute to the damage from toxic effects of heterologous membrane protein expression. TB medium contains higher concentration of yeast extract and tryptone with additional glycerol (0.4% v/v), and the strong phosphate buffer, which make it suitable for long-term growth of cells. Thus, it was selected as the best medium for heterologous expression of hCRs. For pEXP-hCRs, each of the strains showed similar behavior i.e., after induction, the cell cultures reached high densities of 11–13 OD_600_ units, especially in TB medium (data not shown). In most cases, such high cell densities are indicative of a lower protein expression, protein expressed in inclusion bodies, or lower plasmid stability.

### Optimization of inducible hCR expression

In order to optimize high-level hCR production, many variables must be systematically studied including timing, length of induction and culture temperature. To screen the high-yield production strains easily, a rapid and simple method is crucial. We used dot-blot detection with mouse anti-His_6_-tag monoclonal antibody since the expressed proteins all carried the His_6_-tag.

**Figure 4:**
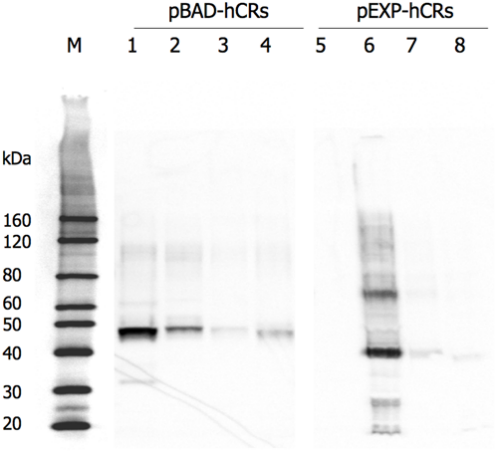
Expression of hCRs detected with Western blot using mouse anti-His_6_-tag monoclonal antibody. SDS-PAGE was performed using NuPAGE 4–12% Bis-Tris gel (Invitrogen, 1× MES buffer). Lane M, His-tagged protein standard size markers (Invitrogen); Lane 1 through 4, proteins expressed by pBAD-hCCR5, hCCR3, hCXCR4 and hCX3CR1 in Top10, respectively; Lanes 5 through 8, proteins expressed by pEXP-hCCR5, hCCR3, hCXCR4 and hCX3CR1 in C41, respectively.

As described in the previous section, pEXP-hCCR3 reached a higher level of expression than did pEXP-hCXCR4 or hCX3CR1, while no expression was detectable for pEXP-hCCR5 (Figure 4). The plasmid stability correlated with their expression level, e.g. higher stability for pEXP-hCCR3 (80%) than for pEXP-hCXCR4 (66%), hCX3CR1 (62%) and hCCR5 (50%).

Expression of pBAD-hCRs in Top10 was similarly studied as for pEXP-hCRs. All the genes were expressed after induction with 0.05% L-arabinose in TB medium at 16–18°C from 2–24 hours. The expression of synthetic hCR genes expressed in pBAD-hCRs with the Trx fusion partner at the N-terminus (Trx-hCRs), are shown in Figure 4. The total expression levels of pBAD-hCRs are higher than those of pEXP-hCRs. Furthermore, the bands (lanes 1–4) show more proteins than those expressed by pEXP-hCRs (lanes 5–8), suggesting more stable and less aggregated expression of pBAD-hCRs in Top10 as compared to the expression of pEXP-hCRs in C41. The plasmids of pBAD-hCRs also show higher stability than pEXP-hCRs, i.e. 75% for pBAD-hCCR5, 93% for pBAD-hCCR3, 60% for pBAD-hCXCR4 and 80% for pBAD-hCX3CR1. Therefore pBAD-hCRs in Top10 were selected for expression of synthetic hCR genes.

The apparent molecular weights of expressed hCRs and Trx-hCRs in SDS-PAGE gel are generally smaller than their calculated molecular weight as shown in Table 1, which is not uncommon for membrane proteins, especially for GPCRs. The membrane proteins samples were mixed with SDS sample loading buffer and incubated at 37°C for 10 minutes before loading. Unlike for samples of soluble proteins, which need be boiled before loading, membrane protein samples cannot be boiled since boiling results in further aggregation. Therefore, membrane protein samples cannot be completely denatured only by SDS, thus have a more compact shape, which tends to faster migration in the SDS-PAGE gel, typically at ∼70–85% of their expected molecular weight [57].

**Table 1:** Parameters of native and reconstructed human chemokine receptors.

	hCCR5			hCCR3			hCXCR4			hCX3CR1		
	Amino Acids	MW(kD)*	pI**	Amino Acids	MW(kD)*	pI**	Amino Acids	MW(kD)*	pI**	Amino Acids	MW(kD)*	pI**
Native	352	40.52	9.21	355	41.04	8.49	352	39.75	8.46	355	40.39	6.74
pEXP3-hCRs	396	45.32	9.10	399	45.84	8.49	396	44.54	8.47	399	45.19	7.29
pBAD-hCRs	491	55.50	8.70	494	56.02	7.07	491	54.72	7.03	494	55.37	6.30

* MW, molecular weight.

** pI, isoelectric point.

### Systematic detergent screening for solubilization of Trx-hCRs

Solubilization from the host cell membrane is absolutely crucial for successfully purification and stabilization of the heterologous membrane proteins [25], [58]. The choice of a suitable detergent for solubilizing an individual membrane protein must be empirically determined. Even for membrane proteins in GPCR superfamily, different detergents have been used for solubilization of different GPCRs [59]. Therefore, a systematic approach for screening a wide range of detergents is essential for obtaining the expressed hCRs.

**Figure 5:**
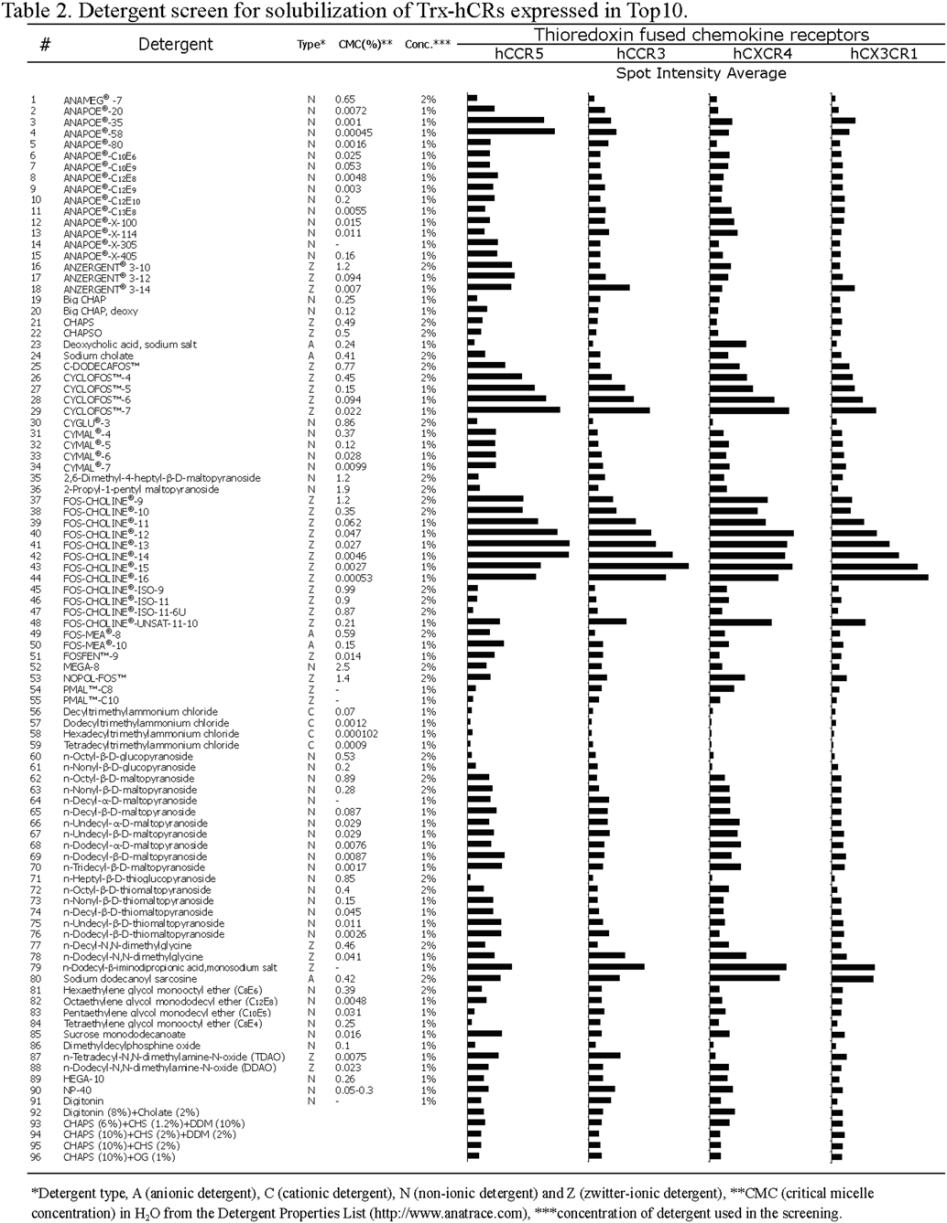
Systematic detergent screens. 96 detergents were systematically screened for their ability to solubilize the Trx-hCRs protein preparation. 96 detergents were selected from the commercial Solution Master Detergent Kit and from literature. Detergents generally are divided into 4 groups depending on their chemical properties, namely, anionic (A), cationic (C), non-ionic (N) and zwitter-ionic (Z). For the majority of detergents, 1% was thus chosen as a standard concentration during the extraction trials, except for detergents with higher CMC, where ∼2% was used. As can be seen from the figure, several clusters of detergents are most effective, particularly phosphocholine (FC) series.

Detergents generally are divided into 4 group depending on their chemical properties, namely, anionic (A), cationic (C), non-ionic (N) and zwitter-ionic (Z) [60]. To investigate the efficacy of various detergents for extraction of Trx-hCRs, 96 detergents were selected from the commercial Solution Master Detergent Kit and from literature. These detergents (Fig. 5) include members of maltosides and glucosides families, such as β-OG, DM and DDM, which have been successfully used to produce crystals; some generally used reagents for solubilization and purification of membrane proteins in many laboratories, such as Digitonin, TDAO, Triton and CHAPS; and some recently developed detergents, i.e. Cyclo-Fos series, Cymal series and Fos-Choline series. In addition, several detergent mixtures were also included in Figure 5 since they had proven effective in solubilization of some GPCRs [59].

The detergent concentrations used during extraction procedures were based on their critical micelle concentration (CMC) values. A concentration above the CMC is required to form micelles. For the majority of detergents, 1% was thus chosen as a standard concentration during the extraction trials, except for detergents with higher CMC, where ∼2% was used. Detergents with much higher CMC (>2%) were not used since their detergent-protein ratios are hard to reach [25], except MEGA-8 (#52), which was chosen for completing the variety of detergents.

Our laboratory has also been studying other GPCRs including human and mouse olfactory receptors since 2004. After extensive screening of a wide ranger of various detergents, Brian Cook found phosphocholine (Fos-) series are most effective for solubilizing and stabilizing human olfactory receptors hOR17-4 that was large-scale produced from mammalian cell [46, Cook, unpublished results]. Cook subsequently found FC14 is optimal for all the hOR17-4 study. Likewise, Liselotte Kaiser and Johanna Graveland-Bikker also found that FC14 is optimal for stabilizing several olfactory receptors produced in wheat germ cell-free system [51] and in yeast Pichia production [unpublished results]. However, it is unknown if FC14 would work equally well in human chemokine receptors. We again carried out a systematic search for optimal detergents

**Figure 6:**
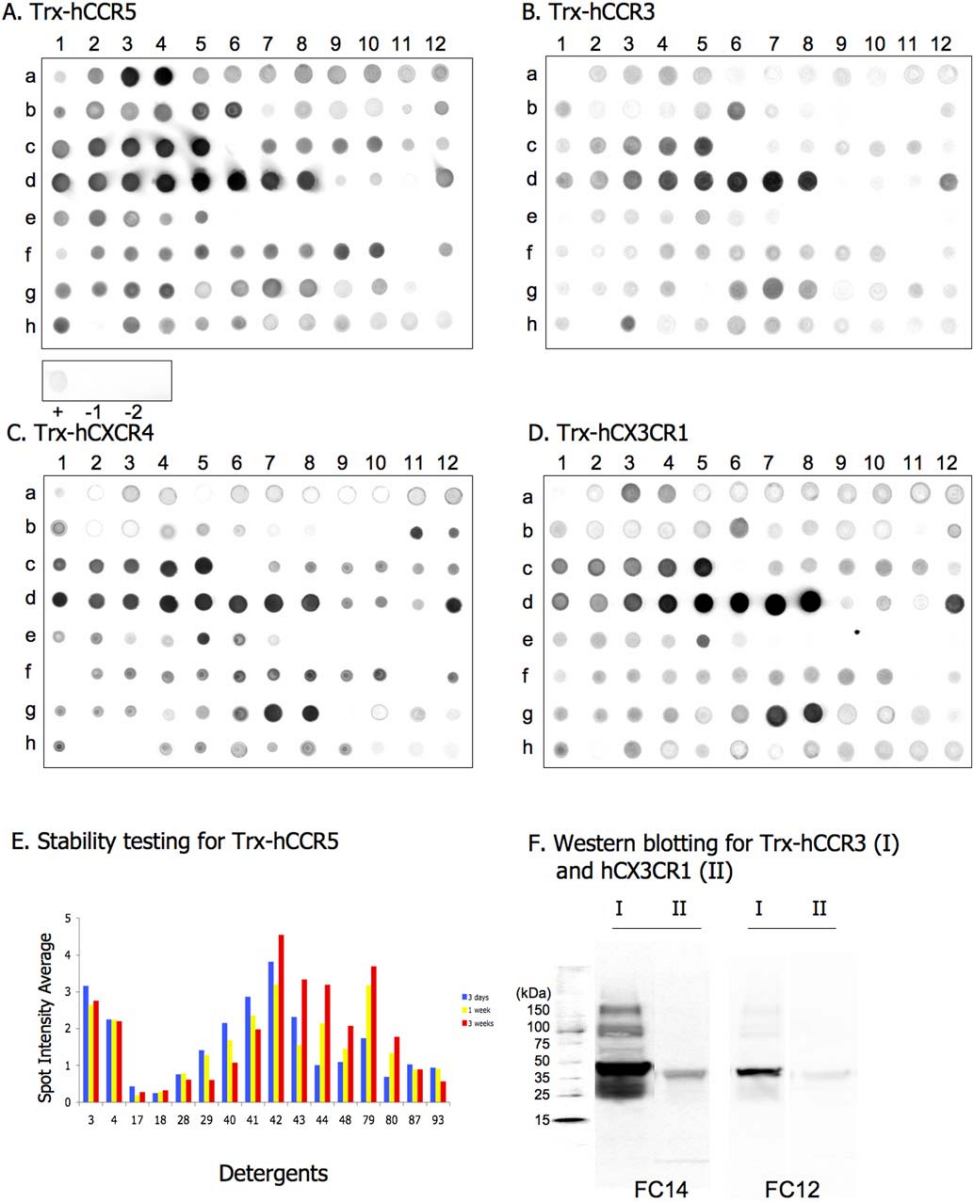
Detergent screening for solubilization of Trx-hCRs expressed by pBAD-hCRs in Top10. A)–D) Effectiveness of the 96 detergents on solubilizing Trx-hCRs from cell lysates, as detected by Dot blot. Immunoblotting analysis was performed using mouse anti-His_6_-tag monoclonal antibody. Each filter has 96 spots (a1 to h12), corresponding to the 96 detergents listed in Figure 5. Controls shown in (A) are positive control (+) using 1∶100 dilution of BenchMark His-tagged protein standard; negative control (−1) was pBAD-hCCR5 in Top10 cultured in the absence of inducer; negative control (−2) was L-arabinose induced sample solubilized without the addition of detergent. The negative controls were processed in parallel with the other 96 samples. E) Long-term stabilization of proteins solubilized in 16 detergents for CCR5 is presented. A comprehensive list of detergents for Trx-hCRs is presented in Figure 5. The detergent effectiveness was carried out at 4°C using Trx-hCCR5 for 3 days, 1 week and 3 weeks, and detection was Dot blot using mouse anti-His_6_-tag monoclonal antibody. Dot blot intensities are shown as a 2-D column chart, and detergent numbers are from the Figure 5. F) Effectiveness of FC14 and FC12 on solubilization of Trx-hCCR3 (I) and hCX3CR1 (II) from cell lysate detected by Western blotting. Protein size standard is shown in the left lane.

Each Trx-hCR expressed by pBAD-hCRs in Top10 was simultaneously solubilized in each of the 96 detergents in a 96-well plate and the extract was examined using Dot blot detection. The extraction of Trx-hCR proteins by detergents was tested on the whole cell lysate (see Method section). The images of dot blots are shown in Figure 6. The higher the solubility it has, the darker the dots are. The average intensity of spots was determined by scanning using FluorChem software. It is presented as a 2D column in Figure 5, with the numbers a1-h12 corresponding to the detergents 1–96.

We carried out analyses by comparing the solubilizing effectiveness of various detergents for Trx-hCR proteins. We identified the most effective detergents (Fig. 5, Fig. 6,). We found two series of zwitter-ionic (Z) detergents, CycloFos series (#26–29) and Fos-Choline series (#37–44) to be most effective of solubilizing all of the Trx-hCRs. Another zwitter-ionic detergents n-Dodecyl-β-iminodipropionic acid, monosodium salt (#79) was also reasonably effective to solubilize Trx-hCRs. Anapoe-35 (#3, termed Brij-35), Anapoe-58 (#4, termed Brij-58) and Anzergent series (#16–18) showed specificity in solubilizing Trx-hCCR5. Maltosides (#62–70), glucosides (#60, 61) and thio-maltosides (#71–76) series were moderately effective for solubilization of Trx-hCRs. Cymal series (#31–34) showed a higher solubilization of Trx-hCCR5 than of other hCRs, including the Cymal-5, which has been used for solubilization of functional CCR5 [61]. Digitonin, CHAPS, DDM and some detergent mixtures, which are of rather general use for GPCR solubilization, show certain capability of isolation of hCRs.

Zwitter-ionic detergents were the most effective for solubilizing hCRs in this system, as they were for solubilizing other GPCRs. For example, DDAO was used for structural studies on rhodopsin, CHAPSO is able to maintain the native structures of CCR5 and CXCR4, and LDAO has been used for solubilizing human leukotriene B4 receptor [62], [63], [64], [65].

**Figure 7:**
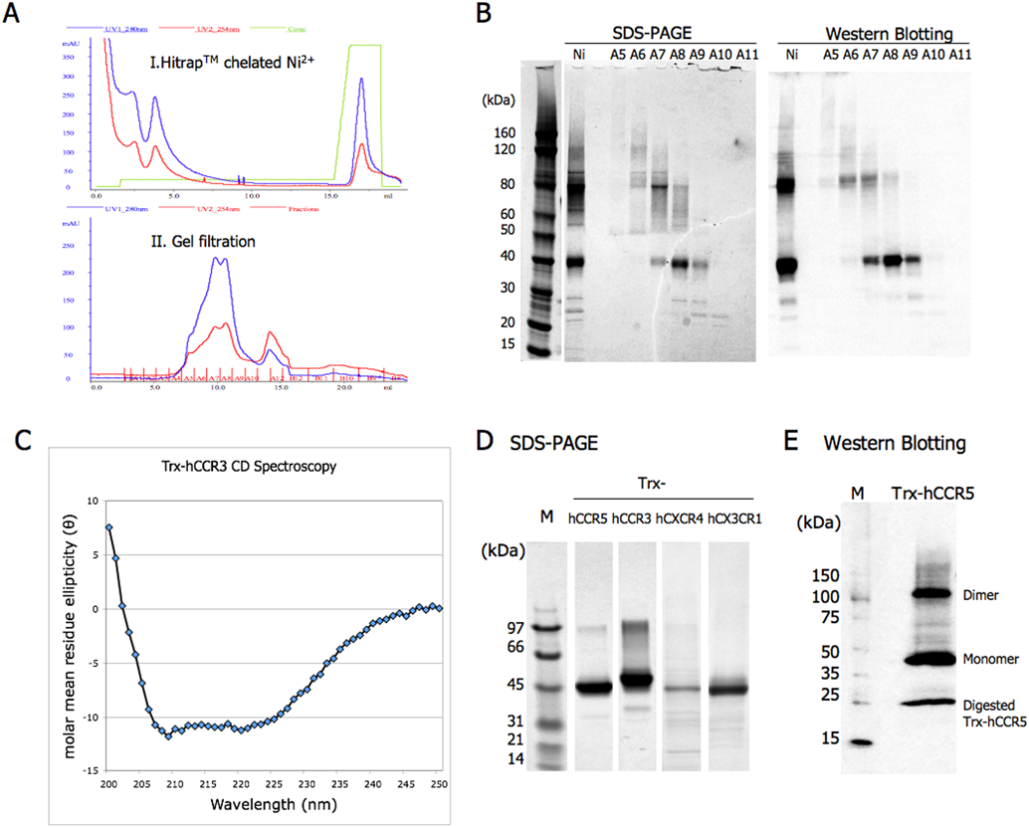
Large-scale purification, identification and studying secondary structure of Trx-hCRs. A) Two-step purification of Trx-hCCR3 from membrane fractions. (I) Ni^2+^ affinity purification of His_6_-tagged proteins using a Hitrap chelating HP 5 ml column. (II) Gel filtration purification using Superdex 200 10/300 GL. Both purification steps were preformed on an ÄKTA Purifier System. B) SDS-PAGE detection and Western blot identification of protein fractions of Trx-hCCR3. One of two duplicates of the SDS-PAGE was transferred onto a nitrocellulose membrane and subjected to Western blot using rabbit anti-human CCR3 monoclonal antibody. Lane Ni: 1/10 dilution of Trx-hCCR3 protein purified by Ni^2+^ affinity column (I); Lane A5 through A11: protein fractions of Trx-hCCR3 corresponding to gel filtration purification (II). All the samples were prepared by mixing 15 µl of protein fractions with 5 µl of SDS Sample buffer (4×). 10 µl of BenchMark His-tagged protein standard was loaded as protein marker. SDS-PAGE was performed in a NuPAGE Novex 4–12% Bis-Tris Gel (in 1× MES buffer, Invitrogen) and stained by Simple Blue SafeStain (Invitrogen). C) Circular dichroism (CD) spectroscopy of purified Trx-hCCR3. Secondary structure of Trx-hCCR3 was analyzed by CD spectroscopy using purified Trx-hCCR3 monomer at 37°C with the concentration of 1 mg/ml in Buffer C. D) All 4 Trx-hCRs were purified and analyzed on SDS-PAGE with size markers and stained using Simple Blue SafeStain. E) Western blot identification of Ni^2+^ affinity column-purified Trx-hCCR5. Fraction of Trx-hCCR5 in the first step of purification was diluted to 0.1 mg/ml and subjected to Western blot detection using rabbit anti-human CCR5 monoclonal antibody. ECL DualVue Western blot Marker was used as protein standard. The 3 bands reveal that different states of Trx-hCCR5 were able to bind Ni^2+^ column. Judging from molecular weight, they may correspond to Trx-hCCR5 dimer, monomer, and truncated Trx-hCCR5, possibly, non-fused hCCR5.

The Fos-Choline series shares the same hydrophilic phosphocholine (Fos-) head group with CycloFos series, but possess a simple hydrophobic tails that likely makes them more effective than CycloFos series. Recently, some members of Fos-Choline series have been used to solubilizing GPCRs, for example, Fos-Choline-16 (FC16) was selected as the suitable detergent for human NK1 receptor (hNK1R) [66]; Fos-Choline 10, 11, 12 (FC10, FC11, FC12) were tested in a high-throughput expression system for membrane proteins, and revealed excellent capabilities for solubilizing membrane proteins, however, at a similar level with maltosides series, NM, DM, UDM and DDM [67], [68]; Fos-choline 14 was used to solubilize human CCR5, but was less effective than Cymal-5 [61]. Fos-cholines 12–16 (#40–44) were the most effective detergents for solubilizing hCRs in our study. Stability of Trx-hCRs (Trx-hCCR5 shown in Figure 7E) was tested in the presence of each of the 16 optimal detergents. Fos-choline14 (#42, FC14) was the most effective of all the tested detergents for stabilizing TRX-CCR5. For the remaining 3 Trx-hCRs, FC13, FC14 and FC15 were effective as well. It is possible that n-Dodecyl-β-iminodipropionic acid, monosodium salt (#79) is also a good choice for CCR5 and CXCR4, and CX3CR1. It is as effective as Fos-choline series, particularly FC14, but it was less effective for Trx-hCCR3.

We used Western blot detection to study the solubilization of Trx-hCRs. Samples of Trx-hCCR3 and hCX3CR1 were solubilized using FC14 and FC12 respectively. FC14 proved more effective for solubilization of Trx-hCCR3 and hCX3CR1 than FC12 (Figure 6F). FC14 was previously found to solubilize and to purify several olfactory receptors including human hOR17-4, mouse mOR23, mS51 and I7 [46]. Furthermore, Fos-choline-14 was used in the crystallization of the *E. Coli* mechanosensitive ion channel MscS for a successful structure [69]. One purified *E.coli* membrane protein in our laboratory has also been crystallized using FC14 and diffracted to 3.6Å resolution (unpublished results). Based on current and the previous detergent optimization, FC14 detergent was selected as standard detergent in all subsequent hCR purifications.

### High-level production of Trx-hCRs

Each of the pBAD-hCRs produced in Top10 was scaled up to 1 liter for high-level production of Trx-hCRs. Washed cell membrane fractions were dissolved in the Solubilization buffer containing 1% FC14 and subjected to ultracentrifugation at 100,000×g for one hour to remove the non-solubilized proteins and collect the supernatant for purification. The purification of Trx-hCCR3 is a simple 2-step process (Fig. 7A). First, the protein was purified by Ni^2+^ chelation chromatography (I), then further purified using size exclusion gel filtration (II). All protein fractions were monitored by SDS-PAGE and Western blot detection (Figure 7B). The proteins purified from the first step include monomer, dimer and higher oligomer of Trx-hCCR3. These species could be further resolved in the gel filtration step.

Trx-hCCR5, Trx-hCXCR4 and Trx-hCX3CR1 were also scaled up and purified, but with a lower yields than for Trx-hCCR3 (Figure 7D). The yields of purified Trx-hCRs finally achieved in this system were ∼1–3 mg/L for Trx-hCCR3, 0.1–0.3 mg/L for Trx-hCCR5, 0.05–0.1 mg/L for hCXCR4 and 0.5–1 mg/L for Trx-hCX3CR1. Individual membrane protein behaves differently, hCCR5 and hCXCR4 showed higher cell toxicity than hCCR3 and hCX3CR1, which may explain the tenfold lower yield of these proteins.

### Secondary structure analysis

Circular dichroism (CD) is one of the best methods to rapidly study secondary structures of proteins, and the far-UV CD spectroscopy from 200 nm to 250 nm can be used to estimate contents of secondary structures. We used the purified Trx-hCCR3 for CD study. The CD profile displayed a typical α-helical curve with ∼40% helical content (Figure 7C). This value is somewhat lower than the theoretical ∼45% (NNPredict at http://ca.expasy.org) and lower than that of typical GPCRs with about 50% α-helical content [65], [70]. It is possible that the contribution to the spectrum from the N-terminal fusion partner HP-thioredoxin may explain this difference. These results suggest that the purified Trx-hCCR3 folded with a reasonable secondary structure as calculated.

### Identification of the purified protein with Western blots using monoclonal antibodies

All the protein fractions in the two-step purification of Trx-hCCR3 were identified using Western blot detection using rabbit anti-human CCR3 monoclonal antibody (Figure 7B). The elution from the Ni^2+^ chelating column purification of Trx-hCCR5 was also identified by Western blot detection using rabbit anti-human CCR5 monoclonal antibody (Figure 7E). However, in addition to that of Trx-hCCR5 (Figure 7E), a smaller fragment showed positive reaction with the hCCR5 monoclonal antibody. We speculated that the small fragment is perhaps a cleavage from the N-terminus of hCCR5 with an apparent molecular weight ∼25 kDa with the abnormal migration for membrane proteins in SDS-PAGE and the monoclonal antibody recognizing the cytoplasmic domain of hCCR5.

### A high-efficiency *E. coli* expression system for high-level production of GPCRs

Biophysical and structural studies of membrane proteins require milligram quantities of proteins. To obtain such an amount is one of the bottlenecks for membrane protein structural determinations [7]. Therefore, significant effort and resources have been invested in obtaining quantity and quality of membrane proteins.

Recently, several human GPCRs were expressed in *E. coli*, but in most cases, at a low level, except for the human NK1 receptor (hNK1R) which was expressed in a large amount, albeit as inclusion bodies [66]. Functional chemokine receptors CCR5 and CXCR4 have been isolated on a small scale using biosensor technology, suitable to be used only for analysis of ligand/receptor interactions [71]. Human adenosine A_2a_ receptor has been functionally expressed in *E. coli* as fusion with periplasmic maltose-binding protein (MBP) at the N-terminus. A yield of 1.5 mg was obtained from 100 g of wet cells [22]. Rat neurotensin receptor is another GPCR expressed in *E. coli* as dual fusions with MBP at N-terminus and Trx at C-terminus. It yield 10 mg from 200 L of culture [23].

Our study is the first report that each of four medically important human GPCR chemokine receptors CCR5, CCR3, CXCR4 and CX3CR1 has been expressed as a membrane-associated protein and purified in milligram amount using synthetic genes expressed in *E. coli* expression systems, as N-terminal thioredoxin fusion proteins. Although no ligand binding assays were carried out to study the function and expression of the Trx-hCRs in membranes, it does not necessarily mean that they are not functional [72]. The Trx-hCRs were detected by their respective monoclonal antibodies, on immunoblots, were highly stable in detergent solution and our CD data indicated that the Trx-fusion CRs were correctly folded. Screens for crystallization have been initiated.

The genes of hCRs encoded using human codon frequency should have no obvious effects on expression in *E.coli*, since it has been reported that even optimization of codon frequency for *E. coli* will not have a significant effect [73], [74]. More likely, the selection of suitable promoters and plasmids is pre-requisite for regulated expression of heterologous genes. The pBAD-DEST49 vector containing the arabinose *ara*BAD operon and the HP-thioredoxin fusion partner are central factors in this system and showed high capacity for expression of GPCRs. The pBAD vectors are particularly suitable for overexpression of membrane proteins, and possess general applicability which is proven by the fact that members of four families of membrane proteins were overexpressed using such vectors and yielded at least 1 mg purified proteins per liter of culture [75]. This is also in part due to the use of the 1,200 fold repressible P_BAD_ promotor, the transcription from which can be regulated in a dose-depended manner by L-arabinose from the concentration of 0.00002% to 0.2% [52].

Overexpression of heterologous membrane proteins fused to a highly expressed soluble protein at the N-terminus has proven very successfully [19], [23], [24], [74], [76]. In the present system, HP-thioredoxin functions as a fusion partner and starts the translation of the target proteins. It may also facilitate to solubilize the GPCR in our system. It remains to be seen if this system can be generalized for other membrane proteins when more and more membrane proteins are produced using this HP-thioredoxin system.

There is evidence that thioredoxin (Trx) possesses cytokine-like and chemokine-like activities [77], which hints at the possibility of specific interactions between the chemokine receptors and their fusion partner Trx. Therefore, we speculate that its function is deeply related to the stability of these chemokine receptor by means of particular interaction helping to stabilize conformation of hCRs. Purification of non-fused hCCR3 expressed by pEXP-hCCR3 in C41 has been carried out, however, the proteins eluted in the Ni^2+^ chelating column were not from the membrane fractions, but from inclusion bodies (data not shown); although the truncated form of Trx-hCCR5 was identified using Western blot (Figure 7E), it could not be separated in the gel filtration step.

Using a low-copy-number plasmid with a moderately strong promoter and lower temperature during production has proven successful for membrane proteins [20]. Grisshammer and Tate reported that low-level bacterial expression could lead to higher degree of integration of the protein to the cytoplasmic membrane of *E. coli*, which is taken as the rate-limiting step for overexpression of membrane proteins [7]. Thus this simple, rapid and easy to scale-up system offers a strategy for high-level production of GPCRs for biophysical and structural studies.

## Materials and Methods

### Materials

Reagents for PCR, TOPO Cloning and Gateway Cloning, vectors, *E. coli* strains One Shot Top10 chemically competent cells (Top10) and BL21(DE3)-STAR-pLysS, DNA Ladder, SDS-PAGE gels and protein standards were purchased from Invitrogen (Carlsbad, CA) unless otherwise noted. OverExpress Competent Cells, including C41(DE3), C43(DE3), C41(DE3)-pLysS and C43(DE3)-pLysS, were obtained from Lucigen (Middleton, WI). All detergents were purchased from Anatrace (Maumee, OH). Nitrocellulose membranes were purchased from Bio-Rad (Hercules, CA). Complete protease inhibitor cocktail tablets, EDTA-free, were purchased from Roche (Mannheim, Germany). Rabbit anti-human CCR3 monoclonal antibody (Ab 32512), rabbit anti-human CCR5 monoclonal antibody (Ab32048) and goat anti-rabbit IgG (HRP) were purchased from Abcam (Cambridge, MA). Mouse His_6_-tag monoclonal antibody and goat anti-mouse IgG/M HRP were obtained from Novagen (Gibbstown, NJ). Protein purification materials were purchased from GE Healthcare Life sciences, Uppsala, Sweden. All common chemicals were obtained from either Sigma (St. Louis, MO) or VWR International unless otherwise indicated.

Liquid growth media used for *E. coli* culture were Luria-Bertani (LB) medium, Terrific Broth (TB) medium and 2YT medium, which were made referring to [78]. Corresponding media plates were made by adding 1.5% agar. The concentrations of antibiotics used in the media and media plates were 100 µg/ml ampicillin, 50 µg/ml kanamycin, and 34 µg/ml chloramphenicol, respectively. Additionally, 0.3 mM isopropyl-1-thio-β-D galactopyranoside (IPTG) or 0.05% L-arabinose was added to make the media plates in the presence of inducer.

Buffers for lysis and solubilization: Lysis buffer, 50 mM sodium phosphate pH 7.8, 200 mM NaCl, 100 mM KCl, 20% glycerol, 10 mM EDTA, 2 mM DTT, 1 mM PMSF, 50 µg/ml lysozyme, 20 µg/ml DNase I; Membrane Wash buffer, 50 mM sodium phosphate pH 7.8, 500 mM NaCl, 100 mM KCl, 20% glycerol, 10 mM EDTA, 1 mM PMSF; Solubilization buffer, 50 mM sodium phosphate pH 7.8, 200 mM NaCl, 100 mM KCl, 20% glycerol, 1% FC14, 1 tablet of protease inhibitor per 10 ml. Buffers for purification: Buffer A (Ni^2+^ chelating column binding buffer), 50 mM sodium phosphate pH 7.8, 200 mM NaCl, 100 mM KCl, 0.02% FC14, 25 mM imidazole; Buffer B (Ni^2+^ chelating column elution buffer), 50 mM sodium phosphate pH 7.8, 200 mM NaCl, 100 mM KCl, 0.02% FC14, 500 mM imidazole; and Buffer C, (gel filtration and purified protein storage buffer) 1×PBS buffer (diluted from 10×PBS buffer, EMD Chemicals, Gibbstown, NJ), 0.02% FC14. Buffers for Western blot and Dot blot: Blocking buffer, 1×PBS, 10% skim milk, 0.1% Tween-20; Wash buffer, 1×PBS, 0.5% skim milk, 0.1% Triton-X-100. Other buffers: DNA loading buffer (6×) was from Novagen; SDS Sample buffer (4×), 250 mM Tris-HCL pH 6.8, 40% glycerol, 8% SDS, 0.004% Bromophenol Blue, 20% Mercaptoethanol, dissolved in milliQ water and frozen at −20°C.

### Methods

#### PCR-based gene synthesis

Human chemokine receptors CCR5, CCR3, CXCR4 and CX3CR1 were selected in the GPCR structural biology program. Protein sequences of the hCRs were obtained from UniProt (Universal Protein Resource), namely human CCR5 (UniProt ID: P51681), human CCR3 (UniProt ID: P51677), human CXCR4 (UniProt ID: P61073) and human CX3CR1 (UniProt ID: P49238), as shown in Figure S1. For PCR-based gene synthesis, the receptor sequence was encoded using the human codon preference using the Dnaworks program (http://helixweb.nih.gov/dnaworks) and parsed into an oligonucleotide set with the following parameters: 45 nt oligonucleotide length, 58°C annealing temperature, 19% of codon frequency threshold, 25 nM oligonucleotide, 10 mM Na^+^/K^+^ and 2.0 mM Mg^2+^. As shown in Figure S1, the encoded genes of hCRs consist of 1056 bp (hCCR5), 1065 bp (hCCR3), 1056 bp (hCXCR4) and 1065 bp (hCX3CR1), respectively. Each gene of hCRs was synthesized as a set of 42 oligonucleotides, synthesized at 50 µmolar scale in 96-well plate format by Integrated DNA Technologies (IDT, Coralville, IA).

PCR-based gene synthesis was performed using a two-step assembly/amplification PCR protocol and designated PCR1 and PCR2. In PCR1, all 42 oligos were mixed and diluted 1∶10 (5 µM for each oligos) in milliQ water as primers and also templates. PCR1 was executed following standard protocol [78], except 58°C anneal for 30 seconds, 72°C extension for 60 seconds, and 35 cycles, which are necessary for achieving full length of gene in PCR2. PCR2 was performed using diluted (1∶100) PCR1 product as template and the two DNA oligos at the ends of the gene as primers, 72°C extension for 90 seconds and 25 cycles. PCR products were separated by 1.5% agarose gel electrophoresis and stained with ethidium bromide (EB). PCR2 products were purified using QIAquick Gel Extraction Kit (QIAGEN) before used in TOPO Cloning, which is a blunt end subcloning used here for inserting the full-length synthetic genes into the pCR-Blunt II-TOPO vector for amplification and DNA sequencing. TOPO Cloning was performed following the manufacturer's protocol. The plasmids with pTOPO-hCR inserts were purified using QIAprep Miniprep kit (QIAGEN) and sequenced.

#### Gene recombination using Gateway Cloning Technology

There are three steps in Gateway Cloning: the *att*B PCR product preparation, the BP Cloning reaction and the LR Cloning reaction (details see Instruction Manual of Gateway Cloning Technology, Invitrogen). The *att*B PCR product of synthetic genes of hCRs were produced by regular PCR using *att*B1-hCR-F (5′- GGGG ACA AGT TTG TAC AAA AAA GCA GGC TTA +24 nt of 5′ terminal forward sequence of hCR) and *att*B2-Histag-hCR-R (5′- GGGG ACC ACT TTG TAC AAG AAA GCT GGG TC TCA ATG GTG GTG ATG ATG GTG +24 nt of 3′ terminal reverse sequence of hCR except for stop code) as primers, with corresponding pTOPO-hCR plasmids as templates. In the *att*B2-Histag-hCR-R primer, His_6_-tag codons were added at the C-terminus of hCRs for expression detection and protein purification. The PCR products were purified and used in the following BP Cloning reaction, which used pDONR-221 vector to make Entry Clones of synthetic genes of hCRs. In the final step (LR Cloning reaction), each gene of hCR was cloned into pEXP3-DEST and pBAD-DEST49 for non-fused and thioredoxin-fused protein expression. All the reactions were performed following the product protocol, and all the Entry clones and Expression clones have been sequenced.

#### Selection of suitable host strains and optimization of cell growth conditions

For selection of suitable host stains, pEXP-hCRs were transformed into *E. coli* strains BL21, C41, C43, C41-pLysS and C43-pLysS, respectively; while for pBAD-hCRs, the *E. coli* strains Top10 was recommended by manufacturer's instruction. For toxicity detection and media type selection of pEXP-hCRs in the five *E. coli* strains, an equal amount of the transformation reactions were spread on LB-agar plates, LB-agar-IPTG plates, TB-agar-IPTG plates and 2YT-agar-IPTG plates, respectively, followed by overnight culture at 37°C. For pBAD-hCRs in Top10, toxicity detection and media type selection were performed in the same way, except that L-arabinose replaced the IPTG as inducer for gene expression. In addition, selection of media types from LB, TB and 2YT was also investigated in liquid media. The protocol was: colonies from LB-agar plates were selected and cultured in 5 ml of LB liquid medium overnight at 37°C with shaking; the next morning, 50 µl of overnight culture was inoculated in 5 ml of fresh LB, TB or 2YT liquid medium, respectively, and to culture was continued at 37°C with shaking while monitoring growth of the cultures by measuring the optical density at 600 nm (OD_600_); at OD_600_ of 0.6∼0.8, the temperature was decreased to 16°C and after 20 minutes, the inducer was added (0.3 mM IPTG for pEXP-hCRs, or 0.05% L-arabinose for pBAD-hCRs). The concentration was monitored every 3 or 4 hours until harvest at 24 hours post induction. All plates and liquid media used here and in later experiments contained 100 µg/ml of ampicillin, except the strains with pLysS were 34 µg/ml of chloramphenicol was added to avoid loss of the pLysS plasmid.

#### Optimization of gene expression

Five colonies from each of the control LB-agar plates (plates I or - in Fig 3A) were selected and separately cultured in 5 ml of LB medium overnight at 37°C with shaking. The following morning, each cell culture was separately inoculated (1∶100) into 3 (for pEXP-hCRs) or 5 (for pBAD-hCRs) tubes containing 5 ml fresh TB medium each. Culture was continued at 37°C with shaking, until the concentration of cell cultures (OD_600_) arrived at 0.6∼0.8. At this point, the temperature of the incubator was lowered to 16°C, and different concentrations of inducer were added to the culture. For pEXP-hCRs in the five strains, three concentrations at 0.1 mM, 0.2 mM and 0.3 mM of IPTG were tested for gene expression, while five different concentrations at 0.01%, 0.02%, 0.05%, 0.1% and 0.2% of L-arabinose were tested separately for the expression of each pBAD-hCRs in Top10. One ml of cell culture was withdrawn from each tube every 4 hours, pelleted at at 5000 g and store at −20°C. These samples were used for detection of expression Dot blot.

Stability of plasmids was also investigated for pEXP-hCRs in C41 and pBAD-hCRs in Top10. For each plasmid, 100 µl was taken from the cultures after 24 hours of expression, diluted in five ten-fold steps from each of which 100 µl was spread on LB-agar plates and LB-agar-ampicillin plates for overnight culture at 37°C. The following morning, plate pairs with about 100 colonies formed were used to calculate the stability ratio of the plasmid.

#### Western blot & Dot blot detection

Sample preparation: stored cell pellets collected from 1 ml of culture were resuspended with 180 µl Lysis buffer. After three cycles of freeze-thaw at −80°C and 42°C, 20 µl of 10% detergent (FC14, unless otherwise stated) was added and mixed well, then, mixtures were incubated for 2 hours at 4°C with gentle shaking. After centrifugation at 16,000 g for 10 minutes the supernatant was removed into fresh tubes for protein expression testing. These samples were stored at 4°C if used within a few days, or at −80°C for future use.

For Western blot detection, 15 µl of each sample was mixed with 5 µl of SDS sample buffer (4×) and kept for 10 minutes at 37°C before applying to SDS-PAGE. Subsequently, the proteins in the gel were transferred onto nitrocellulose membrane following the standard protocol. For Dot blot detection, 3 µl of each of the samples was spotted onto nitrocellulose membranes and let dry at room temperature. All subsequent steps were the same for Western blot or for Dot blot. The nitrocellulose membrane was blocked in 20 ml of Block Buffer for 1 hour at room temperature, followed by washing twice for 5 minutes with 50 ml of PBST (1×PBS, 0.1% Tween-20). For the primary antibody step, the membrane was incubated with 10 ml of 1∶5000 diluted mouse His_6_-tag monoclonal antibody (or 1∶2000 rabbit anti-human CCR3 or CCR5 monoclonal antibody) in Wash buffer for 1 hour, followed by 5 washes for 5 minutes with 50 ml Wash buffer. In the secondary antibody step, the membrane was soaked in 50 ml of 1∶5000 diluted goat anti-mouse IgG/M HRP (or using 1∶4000 goat anti-rabbit IgG HRP if the primary antibody was from rabbit) in Wash buffer for 50 minutes, followed by 5 washes for 5 minutes with 50 ml Wash buffer. Finally, the membrane was developed using ECL plus Western blot Detection Reagents (GE Healthcare) following the manufacturer's protocol and imaged using a FluorChem Image System (Alpha Innotech Corp.). The signal intensities of Dot blot detection (spot intensity average) were quantified using the AlphaEaseFC software of the system.

#### Detergent screening for solubilization of hCRs

Eighty-eight detergents were selected from the Solution Master Detergent Kit (Anatrace). Several detergents with too high CMC values were omitted from the screen. Additionally, HEGA-10, NP-40, Digitonin, and five kinds of detergent mixtures were included the list since they had proven effective in solubilization of other membrane proteins. In total, 96 detergents/detergent mixtures were listed and numbered as they appear in the Solution Master Detergent Kit with the eight additional ones appended as in Figure 5. All the detergents dissolved as 10% except those mixtures.

For sample preparation of the four pBAD-hCRs expressed in Top10, 2 ml of overnight culture was inoculated in 200 ml of fresh TB medium and cultured at 37°C for about 3 hours with shaking, until the concentration reached an OD_600_ of 0.6∼0.8. At this time, the temperature of the incubator was lowered to 16°C and the culture was continued for another 30 min, followed by the addition of 1 ml of 20% L-arabinose (to a final 0.05%) to induce the expression of pBAD-hCRs. After 24 hours of induction, the cells were harvested by centrifugation at 5000 g for 20 min. The cell pellets were resuspended completely with 20 ml of Lysis buffer and distributed into 96 1.5 ml of Eppendorf tubes with 180 µl/tube, numbered from 1 to 96. After 3 times of freeze-thaw at −80°C and 42°C, 20 µl of each detergent (final 1%) was added into the corresponding tube of cell lysate, except for those with higher CMC, where 40 µl of 2% detergent was added to 160 µl cell lysate. The samples were mixed carefully and incubated for 2 hours at 4°C with gentle shaking. Supernatants were collected after centrifugation at 16,000 g and 3 µl of each sample was applied to nitrocellulose membrane in the matrix form shown in Figure 6A from a1 to h12. Detection using Dot blot was performed as described above.

#### Large-scale expression, solubilization and purification of synthetic hCRs

For high-level expression in 1 liter of culture, 10 ml of overnight culture was cooled on ice for 5 minutes and centrifuged at 3000 g for 15 minutes at 4°C. The supernatant was removed and the cell pellets were resuspend in 1 ml of fresh sterile LB medium, and was inoculated into 1 liter of fresh TB medium (containing 100 µg/ml ampicillin) for culture at 37°C with shaking at 220 rpm. When the cell concentration reached about 0.6 (OD_600_), the temperature was decreased to 16°C, and, the inducer was added after an additional 30 min shaking to start the expression of target proteins. The inducer concentrations were 0.3 mM IPTG for pEXP-hCRs in C41, or 0.05% L-arabinose for pBAD-hCRs in Top10. After 24 hours of expression, cells were harvested by centrifugation at 5000 g for 20 minutes, and stored at −20°C for future use.

For collecting membrane fractions, stored cell pellets from 1 liter of culture were resuspended in 50 ml of Lysis Buffer followed by 3 times of freeze-thawing at −80°C and 42°C, and cells were lysed by passing through a French press at 18,000 psi. The cell lysate was centrifuged at 10,000 g for 1 hour to remove cell debris and inclusion bodies, then, ultra-centrifugation was applied for the supernatant at 100,000 g for 1 hour to collect the crude cell membrane fractions. These were resuspended in 20 ml Membrane Wash buffer, followed by another ultra-centrifugation at 100,000 g for 1 hour to collect the washed membrane fractions. Care was taken to resuspend the membrane pellets completely. The next step was solubilization, in which the washed membrane fractions were resuspended in 10 ml of Solubilization buffer containing one tablet of Protease Inhibitor Cocktail, followed by incubation overnight at 4°C with gently shaking. The following morning, the solution was again subjected to ultra-centrifugation at 100,000 g for 1 hour to remove non-solubilized proteins and collect the supernatant for purification.

The supernatants were loaded on a Hitrap Chelating HP 5 ml column, which were charged with chelated Ni^2+^ and equilibrated with Buffer A for the first step of purification. After 15 column volumes (CVs) of washing with Buffer A plus 5% Buffer B (totally 50 mM imidazole), the target proteins were eluted with a linear gradient from 5% to 100% of Buffer B over 2 CVs. The fractions were tested by SDS-PAGE and those containing hCRs were pooled and concentrated using an Amicon Ultra-15 Centrifugal Filter Unit with Ultracel-50 membrane (Millipore). To further improve the purity of the protein, the concentrated protein was loaded onto a Superdex-200 gel-filtration column equilibrated with Buffer C. The peak fractions from the elution were pooled and tested by SDS-PAGE or Western blotting. The concentration of purified proteins was measured with Nanodrop Spectrophotometer (Thermo Scientific).

#### Circular dichroism (CD) detection of secondary structure of Trx-hCCR3

**CD** experiments were preformed on Aviv 202 spectropolarimeter (Aviv Biomedical) with a 1 mm path length cell at 25°C. The purified protein sample came from gel filtration fractions and was concentrated to 1 mg/ml. The CD spectrum was recorded from 200 nm to 250 nm of wavelength with 1 nm resolution and 2 seconds of average time. Buffer C used in the protein purification worked as blank to correct the baseline. Results were expressed as the molar mean residue ellipticity (θ) at a given wavelength.

## Supporting Information

Figure S1 Codon-optimized DNA sequences of human chemokine receptors CCR5, CCR3, CXCR4 and CX3CR1.
(0.04 MB DOC)
