# Supplementary material for: High-Level Production, Solubilization and Purification of Synthetic Human GPCR Chemokine Receptors CCR5, CCR3, CXCR4 and CX3CR1
Source: PLoS One. 2009 Feb 18;4(2):e4509. doi: 10.1371/journal.pone.0004509 (PMC2637981; doi:10.1371/journal.pone.0004509)
Supplement: Figure S1 — Codon-optimized DNA sequences of human chemokine receptors CCR5, CCR3, CXCR4 and CX3CR1. (0.04 MB DOC) [file pone.0004509.s001.doc]

# Supplementary information

**Figure S1.** Codon-optimized DNA sequences of human chemokine receptors CCR5, CCR3, CXCR4 and CX3CR1.

**Human chemokine receptor CCR5:**

**Protein sequence (UniPort ID: P51681)**

MDYQVSSPIY DINYYTSEPC QKINVKQIAA RLLPPLYSLV FIFGFVGNML 50
VILILINCKR LKSMTDIYLL NLAISDLFFL LTVPFWAHYA AAQWDFGNTM 100
CQLLTGLYFI GFFSGIFFII LLTIDRYLAV VHAVFALKAR TVTFGVVTSV 150
ITWVVAVFAS LPGIIFTRSQ KEGLHYTCSS HFPYSQYQFW KNFQTLKIVI 200
LGLVLPLLVM VICYSGILKT LLRCRNEKKR HRAVRLIFTI MIVYFLFWAP 250
YNIVLLLNTF QEFFGLNNCS SSNRLDQAMQ VTETLGMTHC CINPIIYAFV 300
GEKFRNYLLV FFQKHIAKRF CKCCSIFQQE APERASSVYT RSTGEQEISV 350
GL 352

# DNA sequence

ATGGACTACC AAGTCTCTTC CCCCATCTAC GACATCAATT ACTATACCAG 50

CGAGCCCTGC CAAAAGATTA ACGTGAAGCA GATTGCCGCC AGACTGCTGC 100

CTCCCCTCTA CTCTCTCGTG TTCATCTTTG GCTTTGTGGG CAACATGCTC 150

GTCATCCTGA TCCTCATTAA CTGCAAGAGG CTGAAATCCA TGACAGACAT 200

TTACCTCCTC AATCTGGCCA TCTCTGATCT GTTTTTCCTG CTGACTGTGC 250

CTTTTTGGGC TCACTATGCC GCAGCTCAAT GGGACTTTGG CAACACCATG 300

TGTCAACTGC TCACCGGACT GTACTTCATC GGCTTCTTTA GCGGGATTTT 350

CTTTATCATC CTCCTGACAA TTGACCGGTA CCTCGCTGTG GTGCACGCTG 400

TGTTTGCCCT CAAGGCCAGA ACTGTGACCT TCGGCGTGGT CACATCCGTG 450

ATTACATGGG TGGTGGCCGT GTTCGCTTCT CTGCCTGGGA TCATTTTCAC 500

CCGCAGCCAG AAGGAAGGCC TGCATTATAC TTGTTCTAGC CATTTCCCTT 550

ACTCTCAGTA CCAGTTTTGG AAGAACTTTC AGACCCTGAA GATCGTGATT 600

CTCGGACTGG TGCTGCCACT CCTGGTGATG GTCATCTGCT ATTCCGGGAT 650

TCTCAAAACC CTGCTGCGCT GCCGCAACGA AAAAAAGAGG CATCGCGCAG 700

TGCGGCTCAT TTTTACAATT ATGATCGTGT ACTTTCTGTT CTGGGCCCCA 750

TACAACATCG TGCTGCTGCT CAACACCTTT CAGGAGTTTT TCGGACTCAA 800

CAATTGCAGC TCCAGCAACA GGCTGGACCA GGCCATGCAG GTCACTGAAA 850

CTCTGGGGAT GACCCACTGT TGCATTAACC CCATCATTTA CGCCTTCGTG 900

GGCGAAAAGT TTAGGAATTA CCTGCTGGTG TTTTTTCAGA AGCACATCGC 950

CAAGAGGTTC TGCAAGTGTT GCTCTATTTT TCAACAGGAG GCCCCTGAGA 1000

GGGCTAGCAG CGTCTACACA AGGTCCACCG GGGAGCAGGA GATCAGCGTG 1050

GGCCTC 1056

**Human chemokine receptor CCR3:**

## Protein sequence (UniPort ID: P51677)

MTTSLDTVET FGTTSYYDDV GLLCEKADTR ALMAQFVPPL YSLVFTVGLL 50
GNVVVVMILI KYRRLRIMTN IYLLNLAISD LLFLVTLPFW IHYVRGHNWV 100
FGHGMCKLLS GFYHTGLYSE IFFIILLTID RYLAIVHAVF ALRARTVTFG 150
VITSIVTWGL AVLAALPEFI FYETEELFEE TLCSALYPED TVYSWRHFHT 200
LRMTIFCLVL PLLVMAICYT GIIKTLLRCP SKKKYKAIRL IFVIMAVFFI 250
FWTPYNVAIL LSSYQSILFG NDCERSKHLD LVMLVTEVIA YSHCCMNPVI 300
YAFVGERFRK YLRHFFHRHL LMHLGRYIPF LPSEKLERTS SVSPSTAEPE 350
LSIVF 355

## The DNA sequence

ATGACTACTT CTCTCGATAC CGTGGAGACC TTCGGGACCA CCTCCTACTA 50

CGATGACGTC GGACTCCTGT GCGAGAAAGC CGACACCCGG GCTCTGATGG 100

CACAATTCGT CCCCCCTCTC TACTCTCTCG TGTTCACCGT CGGCCTCCTG 150

GGGAACGTCG TGGTCGTGAT GATCCTGATT AAGTATAGGA GGCTGCGCAT 200

CATGACAAAC ATCTATCTGC TCAACCTCGC TATCTCTGAC CTGCTGTTCC 250

TCGTGACTCT CCCATTCTGG ATTCACTACG TCAGGGGCCA TAACTGGGTC 300

TTTGGCCATG GGATGTGCAA ACTGCTGTCC GGCTTCTATC ACACTGGGCT 350

CTACAGCGAA ATCTTCTTTA TCATCCTGCT GACAATCGAC AGGTATCTGG 400

CAATCGTCCA CGCCGTGTTC GCCCTGCGGG CAAGGACCGT CACATTCGGC 450

GTGATTACCT CTATCGTGAC ATGGGGCCTC GCTGTGCTGG CTGCACTGCC 500

CGAGTTTATC TTCTACGAGA CCGAGGAGCT GTTCGAGGAG ACCCTGTGTA 550

GCGCACTGTA CCCAGAAGAC ACTGTGTACA GCTGGAGGCA TTTCCATACA 600

CTCCGGATGA CCATTTTTTG TCTCGTGCTC CCCCTGCTGG TCATGGCTAT 650

TTGCTACACC GGCATCATTA AGACTCTGCT GCGGTGTCCT AGCAAGAAAA 700

AGTACAAGGC CATTAGACTG ATCTTCGTCA TCATGGCAGT CTTCTTCATT 750

TTCTGGACTC CCTACAACGT GGCCATCCTC CTGAGCAGCT ACCAAAGCAT 800

TCTGTTTGGA AACGACTGCG AGCGGAGCAA GCACCTCGAT CTCGTCATGC 850

TCGTCACCGA AGTCATTGCC TATAGCCACT GCTGCATGAA CCCCGTGATC 900

TATGCCTTCG TCGGCGAACG GTTCAGGAAG TACCTCAGGC ACTTCTTCCA 950

TAGACATCTG CTGATGCACC TGGGCAGGTA TATCCCTTTC CTGCCATCTG 1000

AGAAACTGGA GCGGACCTCC TCCGTCTCTC CATCTACTGC TGAGCCCGAG 1050

CTGAGCATCG TCTTC 1065

**Human chemokine receptor CXCR4:**

# Protein sequence (UniPort ID: P61073)

MEGISIYTSD NYTEEMGSGD YDSMKEPCFR EENANFNKIF LPTIYSIIFL 50
TGIVGNGLVI LVMGYQKKLR SMTDKYRLHL SVADLLFVIT LPFWAVDAVA 100
NWYFGNFLCK AVHVIYTVNL YSSVLILAFI SLDRYLAIVH ATNSQRPRKL 150
LAEKVVYVGV WIPALLLTIP DFIFANVSEA DDRYICDRFY PNDLWVVVFQ 200
FQHIMVGLIL PGIVILSCYC IIISKLSHSK GHQKRKALKT TVILILAFFA 250
CWLPYYIGIS IDSFILLEII KQGCEFENTV HKWISITEAL AFFHCCLNPI 300
LYAFLGAKFK TSAQHALTSV SRGSSLKILS KGKRGGHSSV STESESSSFH 350
SS 352

### The DNA sequence

ATGGAGGGCA TTTCCATTTA CACCTCCGAC AACTACACCG AGGAGATGGG 50

CTCCGGGGAT TACGACAGCA TGAAAGAACC ATGCTTCAGG GAGGAGAACG 100

CCAACTTCAA CAAGATTTTC CTGCCAACCA TCTATAGCAT TATCTTCCTC 150

ACCGGCATCG TGGGAAACGG GCTCGTCATC CTGGTCATGG GGTACCAGAA 200

GAAGCTCCGG TCTATGACTG ACAAGTACCG CCTGCACCTC TCTGTCGCTG 250

ACCTGCTGTT CGTCATTACC CTCCCCTTCT GGGCTGTGGA TGCCGTGGCA 300

AACTGGTATT TCGGCAACTT CCTGTGCAAG GCCGTGCATG TGATCTACAC 350

TGTGAACCTG TATAGCAGCG TGCTCATCCT CGCATTTATC AGCCTGGATC 400

GCTACCTCGC TATCGTCCAT GCCACCAACA GCCAGAGGCC ACGCAAACTC 450

CTCGCCGAAA AGGTCGTGTA TGTCGGCGTG TGGATTCCAG CCCTCCTGCT 500

CACTATCCCC GACTTCATCT TCGCTAACGT GAGCGAGGCC GACGACAGGT 550

ATATCTGTGA CCGCTTCTAC CCCAACGACC TGTGGGTCGT CGTCTTCCAG 600

TTCCAGCACA TTATGGTCGG ACTGATTCTG CCCGGGATCG TGATCCTCAG 650

CTGCTACTGC ATCATTATTT CCAAACTGTC TCACAGCAAA GGGCATCAAA 700

AGAGGAAAGC CCTCAAAACC ACCGTGATTC TCATTCTGGC CTTTTTCGCT 750

TGCTGGCTGC CCTACTACAT CGGCATCAGC ATCGACAGCT TTATCCTCCT 800

GGAGATCATC AAGCAAGGGT GTGAGTTCGA GAACACCGTC CACAAGTGGA 850

TTAGCATCAC TGAGGCCCTC GCCTTCTTCC ATTGCTGTCT GAACCCCATC 900

CTGTACGCCT TCCTGGGCGC CAAGTTCAAG ACCAGCGCAC AACACGCACT 950

GACATCCGTC AGCAGAGGCT CTAGCCTGAA GATCCTGTCC AAGGGGAAGA 1000

GAGGCGGGCA CTCTAGCGTG TCCACTGAAA GCGAGAGCAG CTCTTTCCAC 1050

TCCTCC 1056

**Human chemokine receptor CX3CR1:**

# Protein sequence (UniPort ID: P49238)

MDQFPESVTE NFEYDDLAEA CYIGDIVVFG TVFLSIFYSV IFAIGLVGNL 50
LVVFALTNSK KPKSVTDIYL LNLALSDLLF VATLPFWTHY LINEKGLHNA 100
MCKFTTAFFF IGFFGSIFFI TVISIDRYLA IVLAANSMNN RTVQHGVTIS 150
LGVWAAAILV AAPQFMFTKQ KENECLGDYP EVLQEIWPVL RNVETNFLGF 200
LLPLLIMSYC YFRIIQTLFS CKNHKKAKAI KLILLVVIVF FLFWTPYNVM 250
IFLETLKLYD FFPSCDMRKD LRLALSVTET VAFSHCCLNP LIYAFAGEKF 300
RRYLYHLYGK CLAVLCGRSV HVDFSSSESQ RSRHGSVLSS NFTYHTSDGD 350
ALLLL 355

# The DNA sequence

ATGGACCAGT TCCCCGAGAG CGTCACCGAA AACTTCGAAT ATGACGACCT 50

CGCTGAAGCA TGTTACATCG GCGACATCGT GGTGTTTGGC ACCGTGTTCC 100

TGAGCATCTT CTACTCCGTC ATTTTTGCAA TCGGCCTCGT CGGCAACCTG 150

CTCGTCGTCT TCGCCCTGAC AAATAGCAAG AAGCCTAAGT CTGTGACCGA 200

TATCTACCTG CTGAACCTCG CCCTCAGCGA TCTGCTGTTT GTCGCCACCC 250

TCCCATTTTG GACCCACTAC CTGATCAACG AGAAGGGGCT GCATAACGCC 300

ATGTGCAAGT TCACAACCGC TTTCTTCTTC ATCGGGTTTT TCGGATCCAT 350

CTTTTTCATC ACCGTGATCA GCATCGACCG CTACCTCGCA ATTGTGCTGG 400

CCGCAAATTC CATGAACAAC AGGACCGTGC AGCATGGAGT GACCATCAGC 450

CTGGGGGTGT GGGCAGCCGC TATCCTGGTC GCCGCTCCCC AATTTATGTT 500

TACTAAGCAG AAGGAGAACG AGTGCCTGGG CGACTACCCC GAGGTCCTCC 550

AGGAGATTTG GCCTGTGCTG CGCAATGTGG AGACCAATTT CCTGGGCTTC 600

CTCCTGCCAC TGCTCATCAT GTCTTACTGC TACTTCAGGA TCATTCAGAC 650

CCTGTTTAGC TGTAAGAACC ACAAAAAAGC AAAGGCCATC AAACTCATTC 700

TGCTGGTGGT CATTGTGTTT TTCCTGTTCT GGACTCCCTA CAACGTGATG 750

ATTTTTCTGG AAACCCTCAA GCTGTACGAC TTCTTCCCCT CCTGTGACAT 800

GCGGAAGGAT CTGCGGCTCG CTCTGAGCGT GACTGAGACC GTGGCATTTA 850

GCCACTGTTG TCTGAACCCC CTGATCTATG CATTCGCAGG GGAAAAGTTC 900

AGGAGGTATC TGTACCACCT GTATGGCAAG TGTCTGGCCG TGCTGTGCGG 950

GAGGTCTGTC CATGTGGATT TTAGCTCCAG CGAGTCTCAG AGGAGCCGGC 1000

ATGGGTCCGT GCTCTCTTCC AACTTCACCT ATCACACAAG CGATGGCGAC 1050

GCACTCCTGC TGCTC 1065
